# Capturing marine microbiomes and environmental DNA: A field sampling guide

**DOI:** 10.3389/fmicb.2022.1026596

**Published:** 2023-01-12

**Authors:** Nastassia Virginia Patin, Kelly D. Goodwin

**Affiliations:** ^1^Atlantic Oceanographic and Meteorological Laboratory, Ocean Chemistry and Ecosystems Division, National Oceanic and Atmospheric Administration, Miami, FL, United States; ^2^Cooperative Institute for Marine and Atmospheric Studies, Rosenstiel School of Marine, Atmospheric, and Earth Science, University of Miami, Miami, FL, United States; ^3^Stationed at Southwest Fisheries Science Center, National Marine Fisheries Service, National Oceanic and Atmospheric Administration, La Jolla, CA, United States

**Keywords:** microbiome, eDNA, field methods, sampling, molecular ecological methods

## Abstract

The expanding interest in marine microbiome and eDNA sequence data has led to a demand for sample collection and preservation standard practices to enable comparative assessments of results across studies and facilitate meta-analyses. We support this effort by providing guidelines based on a review of published methods and field sampling experiences. The major components considered here are environmental and resource considerations, sample processing strategies, sample storage options, and eDNA extraction protocols. It is impossible to provide universal recommendations considering the wide range of eDNA applications; rather, we provide information to design fit-for-purpose protocols. To manage scope, the focus here is on sampling collection and preservation of prokaryotic and microeukaryotic eDNA. Even with a focused view, the practical utility of any approach depends on multiple factors, including habitat type, available resources, and experimental goals. We broadly recommend enacting rigorous decontamination protocols, pilot studies to guide the filtration volume needed to characterize the target(s) of interest and minimize PCR inhibitor collection, and prioritizing sample freezing over (only) the addition of preservation buffer. An annotated list of studies that test these parameters is included for more detailed investigation on specific steps. To illustrate an approach that demonstrates fit-for-purpose methodologies, we provide a protocol for eDNA sampling aboard an oceanographic vessel. These guidelines can aid the decision-making process for scientists interested in sampling and sequencing marine microbiomes and/or eDNA.

## Introduction

The study of marine microbiomes is critical for understanding global biogeochemical cycles, ecosystem health, and microbial ecology and evolution. Advances in sequencing technology and computational approaches to analyze massive DNA sequence data sets have facilitated important discoveries of marine microbial physiology and interactions (Moran, 2015). Moreover, environmental DNA (“eDNA”) is becoming an important tool for detection of marine macrofauna (e.g., Boussarie et al., 2018; Postaire et al., 2020; Aglieri et al., 2021). However, sampling protocols have yet to be standardized, due largely to an inability to gauge “correct” or “sufficient” data generation, which also depend on factors downstream of sampling such as sequencing strategy. Nevertheless, there are multiple effective methods for collecting eDNA with benefits and disadvantages under various circumstances.

Early oceanographic studies gave microbes little to no consideration. This neglect began to change about half a century ago, when evidence of microbial impacts on photosynthesis and organic matter transformation in the ocean led to new attention on microorganisms (Pomeroy, 1974). The “great plate count anomaly” (Staley and Konopka, 1985) highlighted the shortcomings of cultivation-based assessments of microbial communities, and techniques like microscopy and enzymatic activity assays began to reveal the massive role of marine bacteria in organic matter transformation (Hagström et al., 1979; Fuhrman and Azam, 1982; Azam et al., 1983) and cycling of global carbon (Azam, 1998) and nitrogen (Horrigan et al., 1988; Zehr and Kudela, 2011). With the advent of molecular methods, the discovery of diverse microbial assemblages (Giovannoni et al., 1990; Schmidt et al., 1991) and the recognition of archaea as consistent and important components of marine microbiomes (DeLong, 1992; Fuhrman et al., 1993; Fuhrman and Davis, 1997) further broadened perspectives of microbial evolution and ecology. The extent of marine microbial diversity revealed by molecular methods also highlighted the importance of cultivation efforts, which remain critically important for experimentally validating sequence-based hypotheses. Innovative cultivation strategies have led to the isolation of diverse marine microbial lineages including the ubiquitous heterotrophic bacterium SAR11 (Rappé et al., 2002), the major prokaryotic primary producer *Prochlorococcus* (Chisholm et al., 1992), and the ammonia-oxidizing archaeon *Nitrosopumilus* (Könneke et al., 2005). Nevertheless, it remains challenging to cultivate the marine microbiome, which include members of the Bacteria and Archaea as well as fungi, protists, unicellular phytoplankton, and viruses (Stulberg et al., 2016). Molecular methods, particularly genomics and metagenomics, have thus become a critical window into marine microbiome structure and function and, in some cases, can guide cultivation efforts (e.g., Carini et al., 2013).

**Figure 1:**
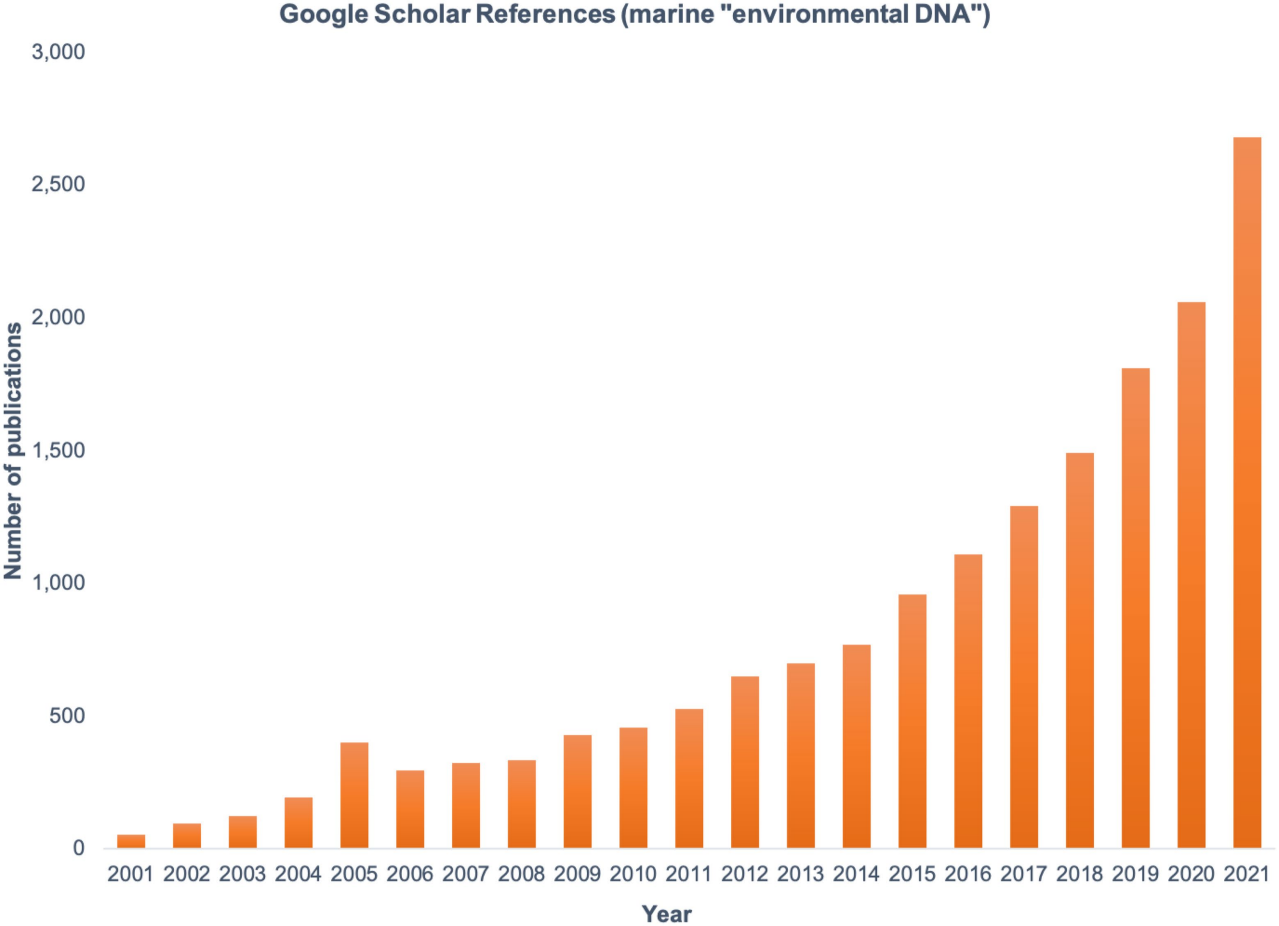
The number of publications found by searching the term marine “environmental DNA” in Google Scholar, shown by year from 2001 to 2021.

Today, the study of marine microbial communities is facilitated by massively parallel DNA and RNA sequencing. The collection of environmental DNA and sequencing of marker genes (for community composition), metagenomes (for functional potential), and metatranscriptomes (for functional activity), collectively known as the field of ‘omics, have become fundamental to improving our understanding of microbial ecology and biogeochemistry in the ocean. Marker gene amplification and Sanger sequencing gave the first insights into the uncultivated diversity of marine prokaryotes (Giovannoni et al., 1990; DeLong, 1992) and eukaryotes (Rappé et al., 1998; Diez et al., 2001; Moon-van der Staay et al., 2001). Metabarcoding studies using high-throughput sequencing provided orders of magnitude more data on planktonic communities compared to clone libraries or denaturing gradient gel electrophoresis (DGGE; Sogin et al., 2006; Stoeck et al., 2010), but functional characterization remained out of reach until the availability of shotgun metagenomic sequencing. The first major biochemical discovery from marine ‘omics data was the gene for proteorhodopsin, an enzyme that allows cells to harvest energy from sunlight without photosynthetic machinery (Béjà et al., 2000). Proteorhodopsins were subsequently found to be widely distributed taxonomically and geographically, altering our understanding of light-based energy flow in the oceans (Rusch et al., 2007; DeLong and Beja, 2010; Olson et al., 2018). Since then, ‘omics work has revealed incredible taxonomic and functional diversity of marine microbiomes (Moran, 2015). Interest in marine eDNA is reflected in the exponential rise in publications using this term (Figure 1) and has expanded to include surveys of higher organisms for biodiversity assessments. As we enter an era of rapidly changing climate and human-driven shifts in marine ecosystems, eDNA and other ‘omics approaches will be critical tools for understanding and predicting community responses from local to global scales.

Even as eDNA-based analyses continue to reveal novel diversity, there are limitations to culture-independent methods. Results can vary widely with differences at every step in the process, from sample collection to preservation to processing and sequencing (Stewart, 2013; McCarthy et al., 2015; Padilla et al., 2015; Torres-Beltrán et al., 2019; Eble et al., 2020). Microbial life has been shown to exist along a physical continuum in the ocean, ranging from truly planktonic to particle biofilms (Stocker, 2012). Homogeneous sampling of this continuum is challenging even within a single study; when methods differ across studies, the chances of equitable sampling further decrease. Moreover, the importance of rare and temporally ephemeral microbial taxa (Galand et al., 2009; Campbell et al., 2011) highlights the need for standardized methods so that results can be compared across time, space, and environmental conditions.

Environmental DNA sequencing has benefited from steep decreases in sequencing costs (Mardis, 2017; Karst et al., 2021) and rapid advances in bioinformatic analysis methods. Amplicon sequencing of rRNA marker genes has been used extensively to characterize marine microbiomes; more recently, improved eukaryotic gene taxonomies now allow accurate surveys of organisms like teleost fish (Miya et al., 2020; Gold et al., 2021) and mammals (Closek et al., 2019). Such metabarcoding studies can provide more information with less sampling effort relative to traditional visual or trawl surveys and combining eukaryotic and microbial community survey data can reveal new linkages among trophic levels (e.g., Djurhuus et al., 2020). However, regardless of their magnitude and accuracy, sequencing data are only as reliable as their source material, i.e., the eDNA.

As the number of marine eDNA studies rapidly increases (Figure 1), the need to share and discuss best practices has grown (Pearlman et al., 2021). Harmonized methods are critical if data are to be compared over space and time for the purposes of making trusted management decisions. Multiple programs are working to establish common guidelines and provide the resources and training to facilitate standardized approaches worldwide (Table 1). Such standardization is especially critical as we consider how global microbiomes will respond to a changing climate and impact broader oceanographic processes.

**Table 1:** A list of programs working toward standardization of marine eDNA sampling and ‘Omics methods.

Program	Geographic scope	Primary aims and/or mission	Program website	Associated protocols/manuals
Marine Biodiversity Observation Network (MBON)	Global	Mission statement: “Foster and coordinate a global community of practice for collecting, curating, analyzing, good management, and communicating marine biodiversity data and related services to the scientific community, policymakers, the public, and other stakeholders.”	https://marinebon.org/	MBON protocols
Ocean Best Practices System	Global	Vision: “To have agreed and broadly adopted methods across ocean research, operations and applications.” Supported by UNESCO, IODE, and GOOS.	https://www.oceanbestpractices.org/	None; documents from Better Biomolecular Ocean Practices (BeBOP) forthcoming.
Bio-GO-SHIP	Global	“To quantify the molecular diversity, size spectrum, chemical composition, and abundances of plankton communities across large spatial, vertical, and eventually temporal scales. This will be achieved through systematic, high-quality, and calibrated sampling of ‘omics, plankton imaging, particle chemistry, and optical techniques as operational oceanographic tools.”	https://biogoship.org/overview/	None
AtlantECO	Atlantic and Southern Oceans	“[…]AtlantECO…aims to develop and apply a novel, unifying framework that provides knowledge-based resources for a better understanding and management of the Atlantic Ocean and its ecosystem services.” European Union-led with collaborators in Brazil and South Africa. Protocols are from *Tara* Oceans.	https://www.atlanteco.eu/	AtlantECO Protocols
Ocean Biomolecular Observing Network (OBON)	Global	“[A] global programme[…]that uses techniques to analyse biomolecules such as DNA, RNA, and proteins (e.g., eDNA analysis, metabarcoding, omics) to greatly enhance coastal and open ocean biodiversity observations.”	https://www.obon-ocean.org/	None; documents forthcoming
European Marine Omics Biodiversity Observation Network (EMO BON)	Europe	Primary aim: “To ensure steady, continuous generation of ‘baseline’ data on biodiversity at EMBRC [European Marine Biological Resource Center] sites following FAIR (Findable, Accessible, Interoperable, and Reusable) data principles.”	https://www.embrc.eu/emo-bon	EMO BON Handbook
The eDNA Society	Japan	Aims: “[F]ostering and developing eDNA science as a discipline that contributes to the human well-being, such as sustainable use of ecosystems and environmental conservation.”	https://ednasociety.org/en/	eDNA Society Manuals
Southern eDNA Society	Australia and New Zealand	Mission statement: “As a society, we aim to promote science and industry collaboration across Australia and New Zealand to advance best practice eDNA methods and adoption in government, private and community sectors.”	https://sednasociety.com/	Protocol Development Guide
Nansen Legacy Sampling Protocols	Polar oceans	“Ten major Norwegian research institutions[…]are sharing their resources, competence and infrastructure in an unprecedented endeavor to provide a cross-disciplinary scientific basis for long-term, holistic, and sustainable management of marine ecosystems and human presence in the northern Barents Sea and adjacent Arctic Ocean.”	https://arvenetternansen.com/	Sampling Protocol Collection
DNAqua-Net	Europe	“The goal of DNAqua-Net is to nucleate a group of researchers across disciplines with the task to identify gold-standard genomic tools and novel eco-genomic indices and metrics for routine application for biodiversity assessments and biomonitoring of European water bodies.”	https://dnaqua.net/	DNAqua-Net Handbook

If there are documents or protocols associated with the program’s efforts, those are provided in the final column. If no documents are provided, the efforts are still in progress or protocols are not (yet) publicly available.

The recognition of this need is illustrated in overarching programs endorsed by the United Nations (UN) Decade for Sustainable Ocean Development that call for harmonized practices, such as the Ocean Biomolecular Observing Network (OBON; Leinen et al., 2022) and Marine Life 2030 (Canonico et al., 2022). There are multiple large-scale efforts to standardize marine microbiome sampling methodologies, and several provide open access resources that can be valuable to those interested in eDNA sampling (Table 1). The Marine Biodiversity Observation Network (MBON) is one of the first biodiversity monitoring programs to perform method comparisons to establish standardized workflows for sampling and analysis of eDNA, including for prokaryotic targets (e.g., Djurhuus et al., 2017; McElroy et al., 2020). Several current efforts feature protocols of the Tara Oceans expedition (Pesant et al., 2015), such as AtlantECO Mission Microbiomes (Pesant et al., 2022) and EMO BON (Santi et al., 2021; Table 1). Others, such as the Bio-GO-SHIP program (Clayton et al., 2022), have expanded to incorporate a subset of these protocols (Pesant et al., 2022).

Nonetheless, a consensus has yet to be reached on a standard sampling workflow, and sequence data continue to be produced at an accelerated pace using a wide range of sampling techniques. The array of available programs working on harmonization is a testament to the importance of and commitment to developing standard practices to ensure that results can be compared and interlaced across studies. For example, the Ocean Best Practices System (Samuel et al., 2021) is a program run by the International Oceanographic Data and Information Exchange and supported by the UN with the stated vision of having “agreed and broadly adopted methods across ocean research, operations and applications” (Pearlman et al., 2019). The project Planet Microbe seeks to associate standardized environmental data with marine ‘omics data sets and has called for large-scale intercalibration efforts to develop community-accepted methods across the full sample-to-data pipeline. Their stated goal of creating “cross-comparable ‘omics datasets[…]to better elucidate global questions on microbial driven biogeochemical processes in the ocean” (Ponsero et al., 2020) captures the growing need for microbiome and eDNA researchers to integrate their results with those of other disciplines and develop a holistic understanding of Earth systems.

## Approach

Literature review is a first step on the path toward standard methods and best sampling practices. This approach is challenging because no one study covers the multitude of possible parameter variations, even for a single molecular target. The variety of targets (viruses to vertebrates) and molecular methods (qPCR to meta-omics) confounds intercomparisons. Extrapolating findings to applications other than those in a study should proceed with caution. Moreover, factors like DNA state (e.g., intra-vs. extra-cellular), decay rates, and transport in the environment are poorly constrained (Andruszkiewicz Allan et al., 2021; Barnes et al., 2021; Jo and Minamoto, 2021; Mauvisseau et al., 2022). Overall, the issues surrounding microbiome and eDNA analyses are complex, particularly for macro-organisms, and a number of reviews and perspectives tackle the challenge of summarizing the state of the science (e.g., Dickie et al., 2018; Harrison et al., 2019; Ruppert et al., 2019; Beng and Corlett, 2020; Kumar et al., 2020; Pawlowski et al., 2020; Bowers et al., 2021; Rodriguez-Ezpeleta et al., 2021).

Although by no means exhaustive, we summarize data from the literature and offer examples from our own experience to guide experimental design. The literature considered here focuses on sample filtration and preservation of marine eDNA using readily available supplies and reagents, although some impactful freshwater studies were included. We address sampling for analysis of prokaryotic and microeukaryotic organisms and include considerations for multi-trophic sampling. We recognize that guidelines are ecosystem specific (Harrison et al., 2019), and recommendations drawn from this analysis may not apply to other marine biomes, such as sediments or host-associated systems. In Supplementary Table S1, we provide an annotated list of studies in which methodological tests were performed. We note where conflicting results necessitate further investigation. As cost is an important consideration for many researchers when designing surveys, we provide a table comparing the cost of different filters and housings to aid study design (Supplementary Table S2).

The main field sampling elements addressed here include water filtration, filter type, sample storage, and DNA extraction. While the latter is rarely performed in the field, it is closely linked to the choice of filter and storage method and thus was an important parameter to include in a guide to overall sampling design. In the final section, we provide an example field sampling workflow that has been used effectively for collecting and preserving marine microbiome eDNA aboard an oceanographic vessel. Filtration based on size fraction is typical for marine eDNA surveys; therefore, eDNA capture methods such as precipitation (Ficetola et al., 2008; Deiner et al., 2015; Eichmiller et al., 2016; Hinlo et al., 2017), centrifugation (Klymus et al., 2015; Eichmiller et al., 2016), or tangential flow capture (Bruno et al., 2017) are not a primary focus. We do not discuss protocols for downstream sample processing such as library preparation, sequencing, bioinformatic data analysis, or standardized metadata. These important topics are outside the scope of this review but are considered elsewhere (e.g., ten Hoopen et al., 2017; Grey et al., 2018; Kelly et al., 2019; Zinger et al., 2019; Mathieu et al., 2020; Berube et al., 2022).

In an effort to build capacity and share knowledge broadly, this overview is appropriate for those new to the field, consistent with goals in programs supported by the UN Decade for Sustainable Ocean Development (Claudet et al., 2020; Canonico et al., 2022; Leinen et al., 2022). We provide both scientific and logistical background to better equip researchers to choose methods appropriate for their needs and circumstances. In accordance with the theme of this special issue, we seek to provide recommendations but do so with humility given the rapid pace of advancement in the field of microbiome science. The aim is to provide a resource for scientists looking to incorporate eDNA into their research or venturing into the field for the first time.

## Logistic and resource considerations

Resources and facilities available for field sampling can vary widely and should be considered during sampling design. Factors like filtration time and sample storage temperature can affect microbiome community composition and diversity results (Rochelle et al., 1994; Oldham et al., 2019), but optimal conditions are often dependent on electrical power, space, and other resources. As with all field work, the protocols for microbiome sampling will differ substantially from those used for sampling in a well-equipped scientific laboratory. In the next sections we will cover the specific aspects of sampling that require particular attention relative to work performed at a typical institutional biological laboratory.

### Clean technique in the field

Microbiome studies are subject to contamination at multiple points along the sample collection and processing pipeline due to the sensitivity of PCR amplification. Standard molecular laboratory practices are designed to reduce the risk of contamination, and systemic contamination appears rare (Sepulveda et al., 2020). However, it can be challenging to protect against contamination during field operations for a variety of reasons. For example, common equipment and supplies available in the lab, such as laminar flow hoods, UV lighting, and supplies of molecular grade water are often unavailable in the field. The level of care needed is dependent on the study; for example, collection of eDNA to detect an invasive species during operations in which the species itself was handled would require the highest level of containment. Moreover, contamination can be mitigated somewhat during sequencing library preparation. Metabarcoding protocols should minimize the number of PCR cycles to prevent exponential amplification of contaminant sequences, while shotgun metagenomic sequencing requires only minimal amplification and is thus less susceptible to low levels of contaminant DNA. In general, microbiome researchers should have a working knowledge of the hygiene practices used in laboratories working with pathogens or radiolabeled chemicals because these specialize in eliminating the spread of trace amounts of target material.

Despite the challenges, simple steps can be applied in field settings to minimize potential contamination. A variety of detailed marine microbiome sampling protocols are available to guide researchers (e.g., Santi et al., 2021; Pesant et al., 2022), with a few key highlights provided here. In general, aseptic practices form the foundation of clean molecular technique. This starts with a clean workspace physically separated from other biological workstations. As with any molecular laboratory, clean gloves and lab coats should be donned upon entering the workspace. Staff that work directly with the target of interest (e.g., fish processing) must ensure that their clothes, shoes, and hair are free of contamination before entering the molecular workspace. The workspace and equipment should be decontaminated prior to initiating work to ensure that exogenous DNA does not contaminate samples. Items coming in and out of the workspace (e.g., coolers, containers) should be minimized and frequent disinfection of the external surfaces can ensure such items do not become a source of sample contamination. A more detailed discussion of common methods for chemical decontamination are provided in the addendum below.

Negative controls should be generated to account for potential sources of contamination according to risk tolerance. Determining the tolerance for risk requires defining the number of samples one is willing to discard if a control shows gross contamination. To minimize costs, a subset of the negative controls should routinely be processed, leaving others as insurance. Although eDNA can travel through the air (Klepke et al., 2022), standard aseptic technique is designed to minimize such contamination. Pilot studies should be conducted to evaluate whether or not collection of routine air blanks is warranted. A variety of reviews can be consulted for additional information (e.g., Goldberg et al., 2016; Mathieu et al., 2020; Sepulveda et al., 2020).

Approaches to minimize sample cross-contamination vary widely, including rinsing with sample water, using various regiments of chemical decontamination, and usage of sterile, single-use consumables. The circumstances are too varied to provide a single recommendation in this regard other than to invest in initial method validation and thereafter practice quality assurance testing routinely. If testing indicates the need for additional work practices or engineering controls, those actions should be taken to ensure robust data generation.

### Hold and filtration times

Water samples should be filtered and processed as soon as possible to prevent changes in microbiome composition and/or eDNA degradation (Rochelle et al., 1994; Oldham et al., 2019). Filtration time may be dictated by logistical issues and/or the stability of the target molecule. If only DNA is targeted, a general guide is to limit filtration time to a maximum of 1 hour. However, there is little rigorous data for filtration of marine water column samples to support specific time limitations. Studies on human microbiome samples (Gorzelak et al., 2015; Song et al., 2016) and eukaryotic eDNA (Thomsen et al., 2012; Maruyama et al., 2014; Yamanaka et al., 2016) have shown changes in DNA yield and composition over relatively short time scales; however, other reports offer conflicting results on microbiome stability (e.g., Lauber et al., 2010). Environmental RNA is more prone to degradation than eDNA (Marshall et al., 2021; Zaiko et al., 2022); thus, shorter filtration times are needed. If RNA is targeted for collection, limit filtration to 20 minutes to provide a practical timeframe to process multiple samples (Zaiko et al., 2022) or 15 minutes to be in accordance with AtlantECO protocols (Pesant et al., 2022).

If immediate filtration is not possible, water should be maintained at <10°C (e.g., in a refrigerator or in a cooler with ice packs) to slow microbial growth. Subjecting the water to freezing temperatures is not recommended, in accordance with guidance provided by the United States Environmental Protection Agency [USEPA; Wymer et al., 2010; United States Environmental Protection Agency (USEPA), 2022] due to the possibility of prokaryotic cell lysis resulting in increased loss of DNA. This recommendation also is supported by results from an eukaryotic eDNA study (Hinlo et al., 2017); however, we recognize that sample freezing is practiced in some laboratories that use eDNA to detect invasive animal species (Hunter et al., 2019). Ideally, validation experiments should be conducted to determine the effect of storage conditions on various taxa. In lieu of application-specific information, we recommend limiting hold times to 6 h, consistent with validated methods to assess recreational water quality using molecular methods [e.g., United States Environmental Protection Agency (USEPA), 2022]. If longer hold times cannot be avoided, the use of a DNA preservative for the intended application should be validated. For example, protocols used by the United States Geological Survey (USGS) employ a combination of 3 M sodium acetate and 95% ethanol to preserve the eDNA of animals in freshwater samples (Ladell et al., 2019).

### Sampling volume

Filtration time and sampling volume co-vary (Zaiko et al., 2022), representing fundamental tradeoffs. Concentrations of target, non-target, and PCR-inhibiting molecules affect sampling design. The amount of microbial biomass and non-microbial particulates affect filtering speed, total collected DNA, and ratio of microbial: eukaryotic eDNA. Coastal water features higher cell concentrations than pelagic water, with factors like proximity to river mouths, time period since last precipitation, and water temperature all affecting microbial biomass and water chemistry. In contrast, pelagic environments, deep water, and oligotrophic biomes such as coral reefs are more likely to feature lower levels of both microbes and particulate matter and may require filtration of higher volumes to capture target species (Kumar et al., 2022). For example, the typical estimate of microbial cell density in the upper 200 m of marine water columns is 5 × 10^5^ cells/ml (Cho and Azam, 1990; Whitman et al., 1998); however, this number can vary widely with depth (Schattenhofer et al., 2009), season (Malone and Ducklow, 1990; Buck et al., 1996; Carlson et al., 1996; Fuhrman and Ouverney, 1998; Whitman et al., 1998; Wigington et al., 2016), and time of day (Gilbert et al., 2010; Weber and Apprill, 2020). Nutrient loads from runoff into the nearshore environment can result in an order of magnitude higher cell concentration than is typical for coastal habitats in both temperate (Palumbo et al., 1984) and tropical (Yeo et al., 2013) locations. Thus, while filtering 100 ml or less may be sufficient from a coastal site next to a river mouth, water from a middle ocean basin may require 2 L or more to capture enough microbial DNA for molecular studies (Kumar et al., 2022).

PCR inhibition causes another fundamental tradeoff in sampling design (Wilson, 1997). Water chemical parameters associated with eutrophication, such as total suspended solids and pH, can impact DNA yield and detection sensitivity (Liang and Keeley, 2013; Tsuji et al., 2017). PCR inhibitors can prevent efficient amplification even in samples with high DNA yields, and inhibitors are often found in systems containing humic acids (Schrader et al., 2012; Cox and Goodwin, 2013; Williams et al., 2017), such as wetland environments and host-associated systems like coral mucus (Sunagawa et al., 2009; Weber et al., 2017). Prior to finalizing a sampling design, researchers should verify the absence of inhibition or the ability to overcome it through DNA dilution or additional purification. For targets available in sufficient quantities, filtration volume can be reduced to avoid later dilution of DNA to combat inhibition. Overcoming inhibition is more challenging for rare targets in samples that contain PCR inhibitors. In one study of an invasive animal species, water filtration across multiple filters and extraction of the combined filters improved eDNA yield; however, extracting from filters individually and combining the resulting products resulted in overall eDNA loss (Hunter et al., 2019). Such results may apply to marine systems but would require verification.

Assuming PCR inhibition is not a limiting factor, a benefit to filtering large volumes of water is that samples will be less sensitive to contamination. One consideration is the availability of sample water, which can be a limited commodity depending on the facilities, equipment, and demands of other teams in the field. In addition, filtering large volumes in a timely manner may require infrastructure that is outside the reach of a typical field program. For example, filtering 20 L of water in 15 minutes or less is feasible aboard *Tara* by employing a dedicated system of sterile carboys, 142-mm filters, and large peristaltic pumps. AtlantECO is developing a filtration kit to address the need to build capacity and promote adoption of this sampling method across the Atlantic Ocean (Pesant et al., 2022).

Our analysis did not reveal a simple answer to the question of the sample volume needed to generate “enough” DNA. Recommendations ranged from 1 L to adequately capture the diversity of phytoplankton (Cermeño et al., 2014) or metazoan eDNA (Stoeckle et al., 2022) to needing 20 L or more to characterize prokaryotic diversity (Pesant et al., 2015). Furthermore, the optimal balance of tradeoffs may depend on the subsequent molecular processing method. For example, the DNA required to capture microbiome diversity (Schmidt et al., 2022) from marker gene amplicon (metabarcoding) or shotgun sequencing likely differs from that needed to successfully conduct a qPCR assay for a specific target (e.g., Andruszkiewicz et al., 2020; Roux et al., 2020; Rourke et al., 2022).

**Figure 2:**
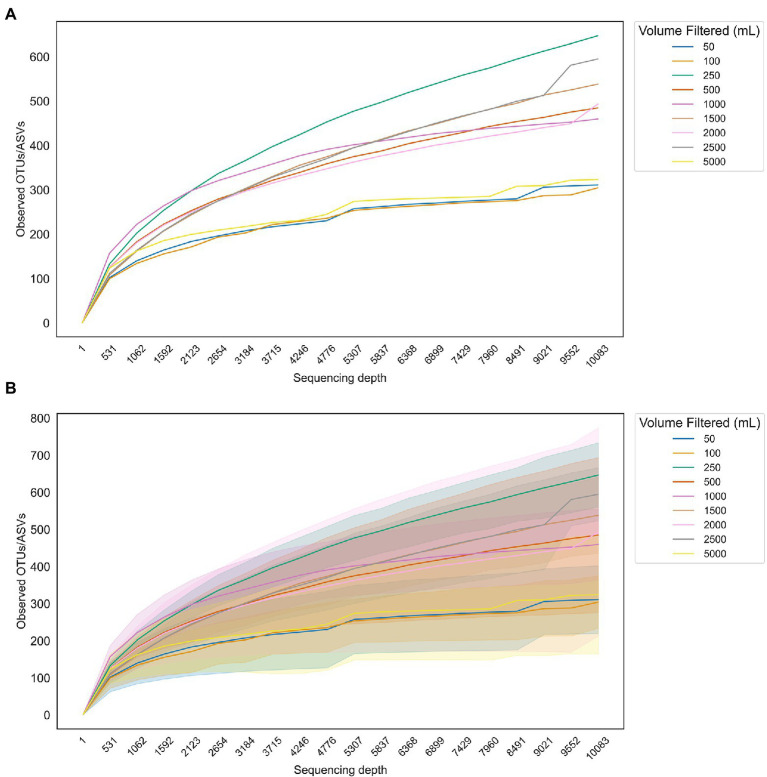
Rarefaction curves showing the number of taxa observed in samples with varying filtration volumes as a function of sequencing depth do not show a clear advantage to filtering larger volumes for downstream sequencing of 16S rRNA gene amplicons. **(A)** Average number of observed taxa for each set of samples corresponding to a different volume of water filtered. **(B)** Curves from **(A)** with standard deviations overlaid.

To run a simple test on the effect of sample volume on observed microbiome prokaryotic diversity, we plotted rarefaction curves (sequencing depth vs. observed taxa, either amplicon sequence variants (ASVs) or OTUs depending on the study) for three publicly available marine 16S rRNA gene amplicons data sets with a range of sample water volumes filtered (Figure 2). The 1 L sample curve shows the highest tendency toward reaching a plateau (Figure 2A), although this may be due to differences in study design; most of the 1 L samples came from the shallow water column and were not pre-filtered (Truelove et al., 2022) while the larger volumes were filtered from a deeper oxygen minimum zone environment with pre-filtration steps (Padilla et al., 2015; Torres-Beltrán et al., 2019). Nevertheless, while community richness varied widely among samples (Figure 2B), there was no apparent advantage to filtering 5 L over 1 L or even 500 ml. Analysis of additional molecular targets was outside the scope of this review; however, a recent paper suggested that up to 40 L of water may be needed for metazoan eDNA targets (Govindarajan et al., 2022).

## Filtration strategy

Filtration strategy varies widely in the literature, with differences in the mode of filtration and in filter pore size, diameter, material, and housing (Supplementary Table S1). These differences reflect considerations for speed, contamination risk, waste generation, demand for clean water, storage space requirements, convenience of subsequent sample processing, costs, desire to maintain past practice, and overall performance. Those attributes are impacted by the type of target(s) to be captured and the concentration of those target(s) relative to non-target components that cause filters to clog and/or inhibit downstream sample processing. Although we do not discuss targeting viral communities in depth, we note that the most widely used protocols involve chemical flocculation of filtrate from the smallest size fraction followed by filtration to capture aggregated viral particles (John et al., 2011; Zhang et al., 2013; Pesant et al., 2022). The ultimate choice of filtration strategy should optimize trade-offs and match the goals and objectives of the eDNA survey.

### Filtration mode

A common way to filter water is *via* vacuum filtration, in which a vacuum pump pulls water through a disc filter. Vacuum filtration using a manifold equipped with multiple filter funnels units is a standard practice in ambient water quality monitoring. If required filtration volumes are small, self-contained sterile filter units exist that are easily transportable, designed for one-time use, and can be attached to a hand pump, making them well-suited to field work on small boats or primitive field stations. However, sample volumes used for eDNA surveys can be >1 L, which may exceed the funnel volume and require an operator to monitor and re-fill the funnel. This is inconvenient and increases the risk of contamination and human error (e.g., mixing of samples) as well as spillage, particularly on a rocking ship. In such cases, a more enclosed system for the sample water is needed, such as carboys fitted with quick-connect vacuum connections.

Another common mode to filter water is *via* peristaltic filtration, in which a peristaltic pump moves water from the sample container through sample tubing and through the filter. Sample volumes are monitored by measuring the amount of water flowing out to waste. Pump speed is easily adjusted allowing finer control of filtration rates than vacuum filtration. Peristaltic filtration can be used with enclosed filters, such as Sterivex™ cartridges, or with membrane filters housed in enclosed containers (e.g., Swinnex™), with filter choices discussed further below. To minimize contamination risk, some approaches attach a sterile serological pipette (e.g., 10 ml) to the tubing to avoid placing the tubing directly into the sample. Approaches to avoid cross-contamination vary and include using new sterilized tubing for every sample, instituting rigorous cleaning procedures between samples, and simply flushing with sample water before sample filtration. All choices balance convenience, cost, and contamination risk. At this time, data appear insufficient to provide clear guidelines other than to ensure that sample contamination is avoided.

Passive filtration has recently emerged as a lower-effort approach to eDNA collection (Bessey et al., 2022; Chen et al., 2022). With this method, filters are directly submerged in the water column and eDNA adheres to a membrane over time, bypassing any water pumping altogether. This approach was recently used to characterize fish community eDNA and was proposed as a method to increase sample replication with subsequent benefits to data analysis (Bessey et al., 2021). Current data are insufficient to assess the potential for passive filtration as a standard method. Nonetheless, the promise of the approach to circumvent filtration provides a clear example of the need to pursue method harmonization while simultaneously embracing innovation.

### Filter type

Filter type determines water flow rate and effective filtration area, and thus is a key component of sampling design. For example, Sterivex™ capsule membranes are available in both polyethersulfone (PES) and polyvinylidene difluoride (PVDF) formats, but PES provides faster water flow rates (Table 2). The pore sizes used depend on the application. For example, 0.45 μm pore size filters are typical in water quality monitoring of fecal indicators [e.g., United States Environmental Protection Agency (USEPA), 2022]. In contrast, marine microbial ecology usually employs 0.2 μm pore size filters to capture the full complement of marine microbial cell sizes (Cottrell and Kirchman, 2004; Kirchman et al., 2007), including picoeukaryotes (Vaulot et al., 2008).

**Table 2:** Example filter types, water flow rates, and filtration areas.

Filter material	Pore size (μm)	Water flow rate (ml/min/cm^2^)^a^
Supor^®^, PES	0.22	26
Durapore^®^, PVDF	0.22	5
Mixed cellulose esters	0.45	>65
Supor^®^, PES	0.45	58
Durapore^®^, PVDF	0.45	26
**Filter type**	**Filter size (mm)**	**Effective filtration area (cm** ^**2**^**)**
Membrane	25	3.4
Capsule (Sterivex)	17	10
Membrane	47	13.8
Membrane	142	127

a at 10 psi, obtained from Pall.com.

Multi-trophic studies are severely challenged by the need to capture the full array of possible cell sizes (Pesant et al., 2022). The challenge is compounded by the fact that metazoan eDNA (e.g., from fish) exists in a range of states, ranging from dissolved to packaged inside an organelle – see Mauvisseau et al., 2022 for a review of this topic. Ultimately, the tradeoffs between filter type, filter time, and sample volume are rooted in the particle size distributions of target eDNA and other non-target suspended particulate matter (Turner et al., 2014; Shogren et al., 2016; Zhao et al., 2021). In the future, perhaps novel capture methods will be devised free from size fractionation. For now, best practices remain reliant on traditional filters. To that end, researchers can conduct tests to estimate the filtration area required (Table 2) for a given set of filtration conditions (e.g., maximum time at constant pressure).

If target cells are particle-associated, a small volume of turbid water may yield sufficient high-quality DNA for the needs of the study (e.g., investigating microbial taxa attached to eukaryotic phytoplankton). Other surveys require more volume to adequately capture the community profile (Kumar et al., 2022). In this case, it can be beneficial to pre-filter and/or size-fractionate the water sample. The term “pre-filter” often refers to removing unwanted large particles before the water passes through the target filter; this method can cut down time required to filter the target volume (Robson et al., 2016), improve PCR amplification due to removal of inhibitors, and reduce variability among replicates (Takasaki et al., 2021). As with the target sample filter, a new pre-filter should be used for each sample to avoid cross-contamination. The pre-filter, with a pore size anywhere from 3 to 20 μm (or even larger, such as a coffee filter or fine mesh), can then be discarded. However, if the pre-filter is preserved, it becomes a second size fraction containing the particle-associated microbial community.

Size-fractionation can both facilitate more effective filtering and be highly informative for comparisons of particle-associated and free-living microbial communities (DeLong et al., 1993; Ganesh et al., 2014; Orsi et al., 2015; Byappanahalli et al., 2021). The research vessel *Tara*, which performs standardized global ocean sampling, including the Tara Oceans expedition from 2009 to 2013, fractionates onto 3 μm and 0.2 μm filters and additionally uses the 0.2 μm filtrate for viral precipitation and final collection on to a 0.8 μm filter (Pesant et al., 2015, 2022). Results from multi-omics analyses provided information on understudied planktonic organisms and their associated microbes that would have been missed with one size fraction (Sunagawa et al., 2020). At the smallest end of the size spectrum, recent work has shown that marine microbes in the Candidate Phyla Radiation (CPR) and the Diapherotrites, Parvarchaeota, Aenigmarchaeota, Nanoarchaeota and Nanohaloarchaeota (DPANN) lineages can have ultra-small cells (<0.1 μm diameter) and would thus evade 0.2 μm filters (Castelle et al., 2018). Methods for capturing such microbial cells have not been widely studied but may include ultracentrifugation (e.g., Boström et al., 2004; Eichmiller et al., 2016) or ethanol precipitation from unfiltered water (Ficetola et al., 2008; Eichmiller et al., 2016; Spens et al., 2017). Jeunen et al. (2019) found that when smaller volumes (500 ml) were filtered, 0.2 μm pore size filters captured significantly more DNA than larger pore size filters, with no differences among filter materials; however, when filtration was performed until filters clogged (up to 5,000 ml), 0.2 μm cellulose nitrate (CN) filters outperformed polycarbonate (PC) and glass fiber (GF) filters with the same pore size in DNA yield. Other studies have shown that GF filters capture more biomass compared to PC or PES filters, but their higher yield may be counteracted by lower sensitivity (Eichmiller et al., 2016; Takahashi et al., 2020).

As with all sampling components, sampling efficacy must be balanced by resource availability. As several studies have shown comparable results in DNA yield and quality among different filters, we do not provide a single recommendation; however, we provide an overview of commonly available filters and their costs in Supplementary Table S2. In the addendum, we describe a sampling protocol we successfully employed aboard an operational fisheries vessel to help illustrate how topics discussed in the main text may manifest in practical situations. We hope the combination of these resources will aid those looking to develop a microbiome sampling routine.

### Filter housing

Filter types can be divided into two major categories: disc and cartridge, or enclosed. Disc filters are flat circular membranes that are generally used with a vacuum filtration setup. After filtration is complete, the filter must be transferred aseptically to a separate container for storage, which poses a risk of contamination. In contrast, enclosed filters (e.g., Sterivex™, Millipore, Billerica, MA) feature membranes in a cylindrical capsule. “Enclosed” cartridges are self-contained throughout the filtration process and can be purchased with luer lock inlets for easy use with syringes and efficient sealing with compatible male adapters. These cartridges are amenable to a peristaltic pump setup. After filtration, the entire capsule is stored while awaiting nucleic acid extraction. This approach typically requires more storage space which needs to be considered in the context of available resources.

Despite offering convenience in the field, Sterivex™ cartridges present challenges for downstream nucleic acid extraction because the filter is bound to the plastic capsule. It must either be physically removed from the housing or reagents must be added to cover the filter area, which can lead to a more dilute nucleic acid product. In any case, most commercially available extraction kits are designed for liquid cultures or tissues that fully dissolve. This circumstance has resulted in a wide array of “standard” modifications to manufacturers’ protocols (e.g., Cruaud et al., 2017; Ushio, 2019; Anderson and Thompson, 2022), further complicating the quest for harmonized practice.

One approach that combines elements of both disc and cartridge filters is a disc membrane housed in a Swinnex™ (Millipore, Billerica, MA). These housings are enclosed like a Sterivex™ during the filtration process and can easily be used with a peristaltic pump, but the Swinnex™ housings are reusable and can be opened to remove the disc. The disc filter-Swinnex™ method is a cheaper and less wasteful alternative to Sterivex™ that still provides the advantages of peristaltic pumping and flexible DNA extraction methods.

Spens et al. (2017) found that Sterivex™ cartridges captured more macrobial (fish) eDNA and fared better at room temperature storage for 2 weeks than membrane filters, particularly with the addition of Longmire’s buffer or ethanol storage buffer. Takahashi et al. (2020) found Sterivex™ cartridges performed better than GF disc filters with low amounts of eDNA, while GF fared better when eDNA levels were high. Their small size, ease of use, and higher resistance to clogging compared to discs make Sterivex™ appealing for many marine microbial eDNA collection applications; however, their higher cost and associated extraction challenges may outweigh their advantages under some circumstances (see section below on DNA extraction).

## Sample preservation

For many field studies, it is impractical or impossible to perform DNA extraction and amplification immediately following sample collection. Safely and efficiently preserving samples is therefore critical for study success. In all cases, filtration procedures should include briefly continuing to operate the pump after all sample water has been filtered to remove excess water before storage. A variety of buffers exist for preventing DNA degradation over time; however, dry preservation can be effective and has the advantage of reducing the required reagents (Majaneva et al., 2018; Sunagawa et al., 2020; Allison et al., 2021). The decision on whether or not to use a buffer will depend on the temperature at which filters can be stored, anticipated number of freeze–thaw cycles, and space capacity in the field. Sample preservation is less critical when target DNA is abundant and quantification is not a primary study goal. Otherwise, the study design may require that all possible precautions are taken to prevent DNA degradation and allow for community assessment.

Immediate freezing of samples after filtration is ideal for nucleic acid preservation. Flash freezing in liquid nitrogen or immediate storage at −80°C are both robust methods of preservation; however, unlike freezers, liquid nitrogen dewars are not prone to electrical issues or breakdowns and are thus preferable for field sampling. Provided immediate freezing is available and filters stay frozen until extraction, samples can be stored dry (i.e., without preservative) with minimal loss of nucleic acid material. Repeated freeze–thaw cycles have been shown to affect host-associated microbiome composition (Sergeant et al., 2012; Gorzelak et al., 2015) and should be avoided. If neither of those approaches are an option, freezing at −20°C as soon as possible offers more protection over 4°C or warmer conditions. However, at these warmer temperatures, additional measures to ensure sample integrity are important, particularly for long-term (>1 week) storage. In one study, the addition of 100% ethanol and room temperature storage for 4 days resulted in DNA yields similar to those from a dry −20°C storage protocol (Hinlo et al., 2017). Other studies found that macrobial DNA remained stable for 1 week in cetyltrimethyl ammonium bromide (CTAB) (Renshaw et al., 2015; Hunter et al., 2019) or up to 150 days at 20°C in Longmire’s buffer (Wegleitner et al., 2015) at room temperature. Longmire’s buffer (3:1 water to buffer) protected bony fish DNA in whole water samples stored for months both frozen and at ambient temperatures (Cooper et al., 2022). Multiple studies found increased yield over time, suggesting that extended lysis buffer storage releases more DNA than a brief incubation.

An increasingly popular method to aid long-term DNA preservation is the addition of silica gel or beads to dry filters. Allison et al. (2021) showed that silica gel preserved DNA integrity for up to 1 year at −20°C and performed better than 95% ethanol at 23°C storage temperature, and Majaneva et al. (2018) found highest consistency in metazoan community composition from filters preserved with silica relative to lysis buffer or ethanol (>99%, molecular grade). This efficacy, along with the important advantage of limiting large volumes of liquid reagents in the field, make silica desiccation a highly appealing option for preservation of environmental DNA.

Along with ethanol, multiple buffers can be used to preserve environmental DNA on water filters with varying levels of efficacy. Common solutions include DNA lysis buffers and reagents containing guanidinium thiocyanate, such as DNAzol^®^ (DN127, Molecular Research Center, Cincinnati, OH), or high levels of ammonium sulfate, like RNAlater™ (Catalog no. AM7021; Thermo Fisher Scientific, Waltham, MA). “Lysis buffer” can include kit reagents such as Qiagen ATL Lysis Buffer or in-house recipes like CTAB, Longmire’s buffer, or sucrose lysis solution (Table 3). Storage buffers can be divided into those that will inhibit DNA extraction and/or purification and must be removed before DNA extraction (i.e., RNAlater, ethanol) and those that can be incorporated into the extraction protocol (lysis buffers). Commercially available buffers like RNAlater and DNA/RNA Shield (Catalog no. R1100-250; Zymo Research, Irvine, CA) can protect all nucleic acids and are often recommended for broadly applicable sampling practices; for example, the European Marine Omics Biodiversity Observation Network (EMO BON) Handbook (Santi et al., 2021).

**Table 3:** Three of the most common preservation buffers and their chemical composition.

Buffer name	Composition	Reference
CTAB	1.4 M NaCl, 2% (w/v) cetyltrimethyl ammonium bromide, 100 mM Tris, 20 mM EDTA and 0.25 mM polyvinylpyrrolidone	Dempster et al. (1999)
Longmire’s buffer	0.1 M Tris, 0.1 M EDTA, 10 mM NaCl, 0.5% (w/v) SDS	Longmire et al. (1997)
Sucrose lysis solution	20 mM EDTA, 200 mM NaCl, 0.75 M sucrose, 50 mM Tris–HCl, pH 9.0	Giovannoni et al. (1990)

In addition to impacting overall DNA yield, there is evidence that storage method can affect taxonomic composition results, presumably due to preferential cell lysis and/or preservation of DNA for certain taxa. Both ethanol and RNAlater yielded communities with lower alpha diversity and higher variability compared to samples preserved with lysis buffer or dried with silica gel (Majaneva et al., 2018; Oldham et al., 2019), and dry preservation provided higher levels of eDNA and more consistent community composition than filters stored with RNAlater in a freshwater lake study (McCarthy et al., 2015). Longmire’s and CTAB lysis buffers have been shown to preserve DNA well at room temperature (Renshaw et al., 2015; Wegleitner et al., 2015) while sucrose lysis buffer performed better than a guanidine thiocyanate buffer (similar to DNAzol^®^) while frozen (Mitchell and Takacs-Vesbach, 2008).

We recommend prioritizing the freezing of samples with minimal delay, and desiccating filters before freezing if possible. If samples will remain at room temperature for an extended period of time or undergo freeze–thaw cycle, storage in Longmire’s buffer or sucrose lysis buffer is an option, depending on the planned DNA extraction protocol.

## DNA extraction

eDNA extraction efficiency from preserved filters depends on the filter type and, to a lesser extent, what type of preservation buffer (if any) was used during filter storage. Extraction protocols can be categorized as highly standardized (e.g., the use of a commercially available kit) or reliant on in-house reagents, which are subject to greater variability among protocols and labs. The reproducibility and accessibility of kits make them an appealing option for comparative studies, large sample numbers, and collaborative efforts. However, there is ample evidence that more involved protocols, such as those with a phenol:chloroform:isoamyl alcohol (25:24:1; hereafter PCI) purification step, result in higher eDNA yields and/or higher levels of eDNA purity (Urakawa et al., 2010; Djurhuus et al., 2017; Schiebelhut et al., 2017; Hunter et al., 2019). These tradeoffs must be weighed while also considering filter types and resource availability, including access to fume hoods and staff willing to work with toxic chemicals. Below we overview these different approaches.

Unlike with enclosed filters (see above), commercial kits are generally available for the extraction of membrane filters with little to no protocol modifications. Many kits provide screw-top vials in which filters can be combined with lysis buffer and stored until extraction, helpfully eliminating the time and effort required to transfer filters between storage and extraction containers. Many studies have produced high-quality microbial eDNA from marine samples using commercial kits. Large scale sampling efforts like Ocean Sampling Day (Tragin and Vaulot, 2018) and the Earth Microbiome Project (Thompson et al., 2017) employed commercial kits that included a bead-beating step, which aids in lysing cells with more resilient cell membranes like Gram-positive bacteria (de Boer et al., 2010) or certain phytoplankton (Mäki et al., 2017). In comparisons of three tested kits offered by Qiagen (Qiagen GmbH, Hilden, Germany), the DNeasy Blood & Tissue kit provided the best results for DNA yield (Djurhuus et al., 2017; Hinlo et al., 2017) and equaled or outperformed the PowerWater kit in metabarcoding data quality and consistency (Jeunen et al., 2019), particularly when modified with a bead-beating step (Djurhuus et al., 2017). Other less commonly used kits described in the literature we reviewed (Supplementary Table S1) include the ZymoBIOMICS 96 DNA/RNA MagBead (e.g., Anderson and Thompson, 2022), Epicentre MasterPure DNA purification (e.g., Geerts et al., 2018), Qiagen AllPrep DNA/RNA mini (e.g., Cruaud et al., 2017), and Presto Mini gDNA (e.g., Jeunen et al., 2019), but we found too few studies describing the performance of these kits to comment further.

The two most common non-commercial approaches for extracting DNA are protocols using CTAB or PCI; in some cases, both reagents are used (Needham and Fuhrman, 2016; Hunter et al., 2019). These protocols rely on chemical cell lysis and generally do not include a bead-beating step, although one can be added (e.g., Urakawa et al., 2010; Biller et al., 2018). A PCI protocol was used for high-throughput sampling (>600 marine microbial metagenomes) in the bioGEOTRACES study (Biller et al., 2018) in a testament to its efficacy and lower cost. A combination CTAB/PCI was effectively used to extract microbial and phytoplankton DNA for a multi-trophic metabarcoding study (Needham and Fuhrman, 2016). These protocols can be particularly useful for enclosed or capsule filters, which can be challenging to integrate with kits. However, they rely on hazardous chemicals and require significantly more bench time than kits, limiting their usefulness for high-throughput sample processing. Several studies have found that PCI yielded higher microbial and macrobial DNA copy numbers (Urakawa et al., 2010; Renshaw et al., 2015; Djurhuus et al., 2017; Schiebelhut et al., 2017) and higher DNA purity (Urakawa et al., 2010; Schiebelhut et al., 2017) compared to kits. Other studies found higher detection rates for some, but not all, kits compared to PCI (Deiner et al., 2015). Due to better performance but higher effort, PCI protocols are recommended for rare taxa and/or if the number of samples to be processed is relatively small.

To extract nucleic acids from enclosed filters such as Sterivex™, storage buffer must be completely removed from the cartridge unless it can be integrated into the extraction protocol (e.g., Ganesh et al., 2014; Padilla et al., 2015). Removal can be performed by flushing with air using a syringe or a vacuum manifold. Lysis buffer must then be added to the cartridge, or the filter can be removed from the plastic housing. Cruaud et al. (2017) found that removing the Sterivex™ housing, cutting up the filter, and adding filter pieces to tubes with lysis buffer yielded 4 times as much DNA as an internal extraction protocol and produced similar alpha diversity and community composition results. Kawato et al. (2021) also recommended this method for detection of deep-sea fish. However, this process is susceptible to contamination, negating one of the main advantages of the enclosed cartridge.

Alternatively, beads can be added to the Sterivex™ cartridge housing. This mechanical lysis step improved microbial community DNA yield over both an internal extraction protocol without beads and a protocol based on opening the cartridge, while maintaining the enclosed filter environment (Ushio, 2019). Anderson and Thompson (2022) adapted this protocol in several useful ways, including adding beads after sample collection, optimization of bead composition to maximize recovery of hard-to-lyse organisms, and demonstrating high-throughput extractions performed on a magnetic bead-handling robot [KingFisher™ Flex Purification System (Thermo Fisher Scientific, Waltham, MA)].

All the protocols described above are well suited for subsequent manual benchtop library preparation and short read sequencing, which remain the predominant approaches for metabarcoding or metagenomic sequencing. However, technological advances are rapidly changing the DNA sequencing landscape and deserve consideration. Long read sequencing, which can be performed by PacBio^®^ or Oxford Nanopore Technology^®^ platforms, requires higher molecular weight DNA than short read platforms like Illumina (Jones et al., 2021). Thus, protocols designed to minimize shearing (e.g., Mayjonade et al., 2016; Russo et al., 2022) should be considered if long read sequencing is planned. Trigodet et al. (2022) found that column-based commercial kits with enzyme modifications were equally as, if not more, effective than PCI protocols for generating high-quality long read sequences. In addition, robotic sampling and processing instruments, such as the KingFisher™ Flex Purification System mentioned above, can reduce the time and effort required to process eDNA samples (Marotz et al., 2017; Anderson and Thompson, 2022). Autonomous samplers, like the Monterey Bay Aquarium Research Institute Long-Range Autonomous Underwater Vehicle containing an Environmental Sample Processor (3G ESP-LRAUV), can filter marine water *in situ* and preserve filters equivalent to traditional shipboard sampling for eDNA analysis by qPCR (Yamahara et al., 2019) or DNA metabarcoding (Truelove et al., 2022). These and similar technological advances indicate the field of marine microbiology has entered an exciting new era of discovery. Many of the field sampling considerations presented in this review may thus become irrelevant in the not-too-distant future. However, until such instruments become mass-produced and cost-effective, most labs will continue to rely on manual sampling and extraction protocols.

## Discussion

The need to standardize sampling approaches for marine microbiomes and eDNA is widely recognized (Canonico et al., 2019; Pearlman et al., 2019; Hörstmann et al., 2021; Samuel et al., 2021). The wide ranges of physical eDNA states, applications, and test conditions available in the literature make finding consensus difficult. The quest for consensus is further confounded by the desire to maintain consistent methods once a particular study or time series has begun, a limited ability to unify the large community of researchers spread across the globe, and the fact that most marine eDNA surveys try to develop a fit for purpose protocol while actually attempting to meet multiple goals (e.g., a “microbes to mammals” approach).

Despite the surplus of data, protocols, and guidelines available, pilot studies should be designed, executed, and analyzed prior to committing to a protocol. Therefore, programs need to budget adequate time and money to verify that protocols meet the needs of the study. Protocol development should be undertaken in the context of environmental variability with the aim of elucidating which parameters are truly important to control. For example, investing in more PCR technical replicates is not warranted if the sample collection volume is insufficient for the purposes of the project.

Despite the lack of consensus on microbiome sample collection and processing methods, we recognize certain overarching guidelines here. Rigorous decontamination of work surfaces and equipment, sample replication, and controls included throughout the sample collection and processing workflow are baseline practices. The need to rapidly collect, filter, and freeze samples with minimal delay is balanced by the requirement to obtain sufficient DNA to detect and characterize the species of interest. Partnerships between marine science and industry should be pursued to develop fast and reliable means to collect, preserve, and reproducibly extract large quantities of high-quality DNA from aquatic samples.

We recognize that differences in microbial and eukaryotic DNA prevalence (ubiquitous vs. patchy), concentration (high vs. low), and state (associated with different size fractions) lead to conflicting approaches for ideal sampling strategies; nevertheless, it is possible to design a versatile protocol that allows multiple sampling goals to be achieved. Ideally such a design can be implemented on a minimal budget and executed by readily available crew with little specialized training as they transverse waters from coastal to open ocean. An overarching authority with regard to best practices is needed but it is unclear how it might emerge. Emerging efforts such as the Better Biomolecular Ocean Practices (BeBOP; Table 1) are promising in this regard, although a disconnect in time scales between those processes and the pace of technological advancement is a concern (Trujillo-González et al., 2021). We recommend continued efforts in this area with an emphasis on including a diverse array of marine microbiome researchers who collectively hold a vast amount of knowledge, much of which remains anecdotal or unpublished.

The ultimate goal of harmonization efforts is to bring the field closer to a consensus on best practices (Pearlman et al., 2021) for sampling marine microbiomes. Harmonized approaches are critically needed as the pace of eDNA studies accelerates and the call to understand microbiome responses to climate change increases (Cavicchioli et al., 2019; Tiedje et al., 2022). Method harmonization will enable the current patchwork of observations to be stitched into a global network of observations to produce baselines by which ecosystem impacts can be accessed. Producing data that is interoperable over space and time is the first step to building a trusted time series upon which to base management decisions.

## Addendum

### Example protocol: eDNA sampling during a fisheries survey

**Figure 3:**
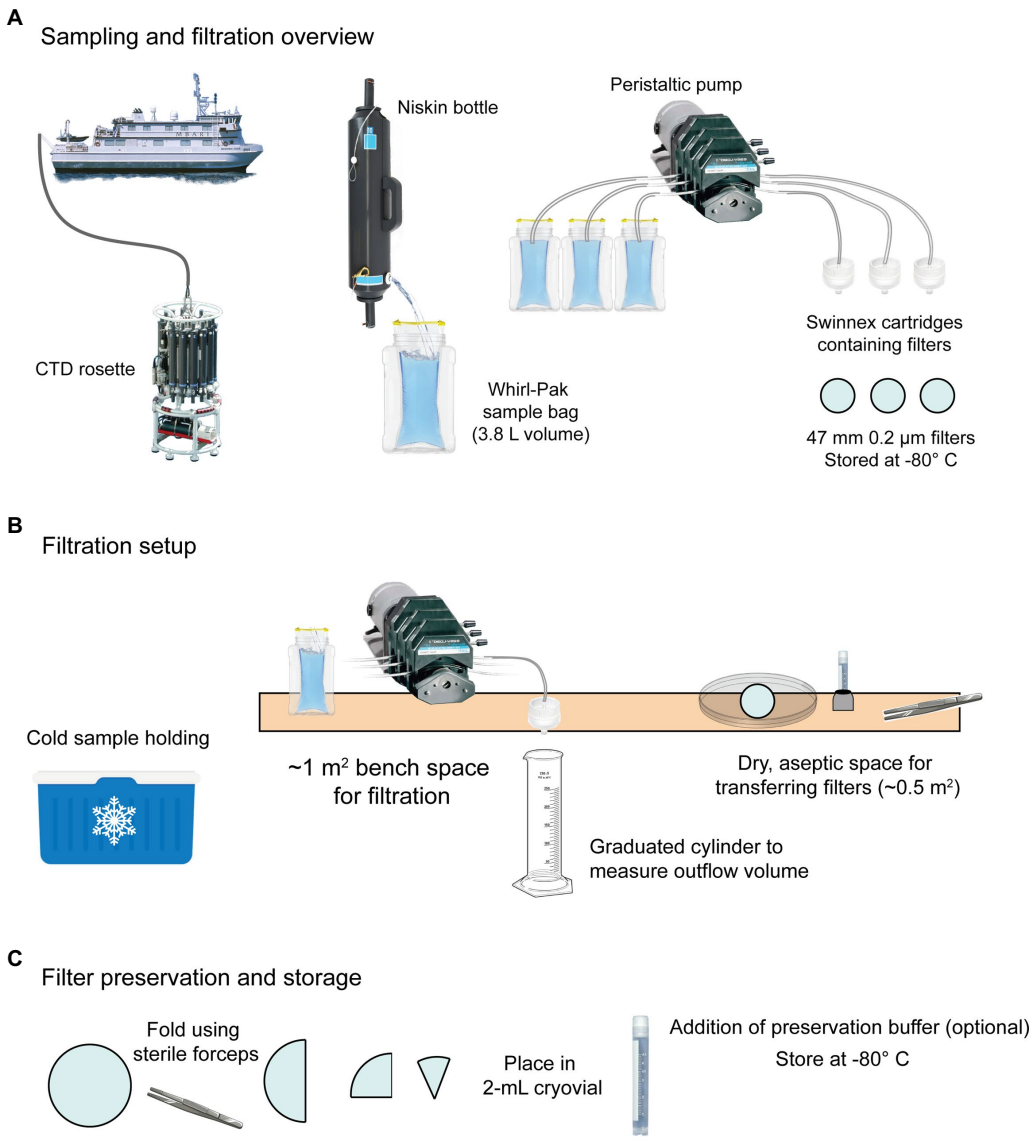
Example shipboard sampling workflow for eDNA based on MBON recommendations. **(A)** Water is collected using Niskin bottles mounted on a CTD rosette. Once aboard, water is transferred to sterile Whirl-Pak^Ⓡ^ bags and filtered using a peristaltic pump and membrane filters housed in Swinnex cartridges. **(B)** Whirl-Paks with sample water are stored cold (4°C) until filtration, which is performed on a bench with a peristaltic pump and Swinnex™ housed disc filters (47 mm diameter). Outflow is measured using a graduated cylinder and the filtration duration and volume is recorded. Filters are transferred from the housing to a 2-ml cryovial for storage. **(C)** Filters are loosely folded in cryovials. Preservation buffer can optionally be added at this point. Cryovials should be immediately stored at −80°C or in liquid nitrogen.

Here we describe a marine eDNA sampling protocol to illustrate designing a fit for purpose “best practice” to balance the advantages and disadvantages described above. This protocol (Figure 3) is based on the experimental goals and available resources of our particular field sampling vessel and is based on the MBON protocols.^1^

#### Goals

We collected marine eDNA in conjunction with midwater trawls conducted during the annual NMFS Rockfish Recruitment and Ecosystem Assessment Survey (RREAS). eDNA sequencing and analysis included metabarcoding of microbial (16S and 18S rRNA) and eukaryotic/metazoan (12S rRNA and COI) gene amplicons, as well as shotgun metagenomics.

#### Field sampling facility

The eDNA lab aboard the vessel was separate from the main wet lab, which was frequently exposed to high levels of fish and microbial DNA from the trawls. A −80°C freezer was situated in a separate part of the ship. There was no Milli-Q water system available, but we collected freshly distilled water from the vessel’s distillation system in the engine room using containers cleaned thoroughly with bleach and Milli-Q water ahead of the cruise. Sample water was collected using 10-L Niskin bottles mounted on a CTD rosette.

#### Preparation

Peristaltic pumps were set up on a bench space that was thoroughly cleaned with a 5%–10% bleach solution. Tubing was rinsed with ~500 ml bleach by running the pump, followed by a rinse with Milli-Q water. When possible, up to 1 L of Milli-Q water was used to thoroughly remove bleach; however, to compensate for the Milli-Q water limitation we incorporated a rinse with 1–2 L sample water per tubing (see below).

#### Notes regarding decontamination protocols

Complete and efficient decontamination of surfaces can be performed with various chemicals, with two common approaches described here. Sodium hypochlorite (bleach) has long been recognized as a low-cost, highly effective way of degrading DNA (Prince and Andrus, 1992). Typical protocols call for a 10% bleach solution; however, it is a common misconception that this solution refers to a 1:10 dilution of commercially available household bleach products, which are usually 6%–8% sodium hypochlorite (Santi et al., 2021). In fact, final w/v of sodium hypochlorite should be 1%–5%, requiring at most a 1:8 dilution with most commercial bleach products (Kemp and Smith, 2005; Goldberg et al., 2016; Wilcox et al., 2016). To fully decontaminate surfaces and equipment, bleach should be applied and sit for ~15 min. Complete removal of the bleach solution is then crucial to prevent residual sodium hypochlorite in the water from causing sample degradation. A thorough rinse of all tubing and filtration equipment with sterile water (fresh or saline) or with extra sample water after application of chemicals for decontamination is recommended. High bleach concentrations can be challenging to remove, especially if left sitting for a long time; we therefore recommend a 1% w/v bleach solution left for at most 20 min on any surface.

Many protocols suggest RNase AWAY™ (a sodium hydroxide solution) or hydrochloric acid in addition to or as an alternative to bleach (e.g., the MBON water sampling protocol). For small volume applications (e.g., sterilizing forceps) RNase AWAY™ is highly effective but due to its substantially higher cost is not a practical reagent for large-scale decontamination. These solutions tend to be gentler than bleach on stainless steel surfaces. However, all chemicals are toxic and require cautious handling.

#### Sampling

Water from Niskin bottles was collected in sterile 3.8-L Whirl-Pak bags by direct transfer (allowing a “clean stream” to run briefly before beginning collection). From each sampled Niskin, a separate 2-L Whirl-Pak bag was filled to provide water for rinsing. Bags were stored in a cooler with wet ice during the filtration process. Before filtration began, bags were inverted 3–5 times to thoroughly mix sample water. The first round of pumping was done without attached filters and using water from the 2-L “rinse bags” to rinse the tubing with ~1 L water from the sample Niskin bottle. To capture eDNA from sample water, 47-mm, 0.22-µm nitrocellulose filters in a Swinnex housing were attached to the tubing outflow end and a new serological pipet was attached to the intake end. The pipet end was placed in the sample bag (with tubing remaining outside) and the pump was run until 2 L of water was filtered or the filter clogged, whichever came first. Before stopping the pump, air was briefly run through the tubing to dry the filter.

#### Sample preservation

When filtration was complete, the pump was turned off and the Swinnex housing was disconnected from the tubing. The housing was opened and the filter was folded with sterile forceps and transferred to a cryovial. This container was placed inside a secondary container (e.g., a 250-ml Whirl-Pak bag) containing silica beads. Biological replicates were stored together in one bag, and full bags were transferred to the −80°C as soon as possible. Samples were stored on the ship and transferred on dry ice to the laboratory upon mission completion, where they were stored at −80°C until processed in bulk at a facility capable of high-throughput DNA extraction.

## Methods

Figure 1 was generated by searching Google Scholar for the terms ‘marine “environmental DNA”’ so that eDNA from a marine environment would likely be a major component of the study. The filter was set to limit results from each year sequentially and results were plotted in Microsoft Excel.

Alpha rarefaction curves were generated from three publicly available marine water column 16S rRNA gene amplicon data sets generated by the following three studies: 1. Truelove et al. (2022), 2. Padilla et al. (2015), and 3. Torres-Beltrán et al. (2019). Raw sequences from [1] and [2] were run through DADA2 in QIIME2 with the following parameters: The processed OTU table from [3] was combined with the processed sequences from [1] and [2] and rarefaction curves were generated from the resulting feature tables using ‘qiime alpha rarefaction’ with a maximum sequence depth of 10,083 and 20 sub-sampling steps. The resulting rarefaction table was exported as a csv file and run through a custom Python script to generate rarefaction curves with and without standard deviations for each line. The script is available at the first author’s GitHub repo: https://github.com/nvpatin/Amplicon-visualizations.

## Author contributions

NP performed the analyses and created the figures. NP and KG wrote the manuscript. All authors contributed to the article and approved the submitted version.

## Conflict of interest

The authors declare that the research was conducted in the absence of any commercial or financial relationships that could be construed as a potential conflict of interest.

## Publisher’s note

All claims expressed in this article are solely those of the authors and do not necessarily represent those of their affiliated organizations, or those of the publisher, the editors and the reviewers. Any product that may be evaluated in this article, or claim that may be made by its manufacturer, is not guaranteed or endorsed by the publisher.

## Footnotes

^1^ https://mbari-bog.github.io/MBON-Protocols/WaterFilteringProtocol.html
